# Association Between Preonset Anti-hypertensive Treatment and Intracerebral Hemorrhage Mortality: A Cohort Study From CHEERY

**DOI:** 10.3389/fneur.2022.794080

**Published:** 2022-03-07

**Authors:** Yan Wan, Hongxiu Guo, Jing Shen, Shaoli Chen, Man Li, Yuanpeng Xia, Lei Zhang, Zhou Sun, Xiaolu Chen, Jiang Chang, David Wang, Quanwei He, Bo Hu

**Affiliations:** ^1^Department of Neurology, Union Hospital, Tongji Medical College, Huazhong University of Science and Technology, Wuhan, China; ^2^Department of Epidemiology and Biostatistics, Key Laboratory for Environment and Health, School of Public Health, Tongji Medical College, Huazhong University of Science and Technology, Wuhan, China; ^3^Neurovascular Division, Department of Neurology, Barrow Neurological Institute/Saint Joseph Hospital Medical Center, Phoenix, AZ, United States

**Keywords:** intracerebral hemorrhage, hypertension, hematoma volume, mortality, CHEERY study

## Abstract

**Introduction:**

Hypertension is the most prevalent risk factor for intracerebral hemorrhage (ICH). In this study, we investigated whether preonset anti-hypertensive therapy could affect the outcomes of ICH.

**Methods:**

This was a retrospective cohort study. A total of 3,460 consecutive patients with acute first-ever ICH from 31 recruitment sites were enrolled into the Chinese cerebral hemorrhage: mechanism and intervention (CHERRY) study from December 1, 2018 to November 30, 2020, and 2,140 (61.8%) with hypertension history were entered into the analysis.

**Results:**

Only 586 patients (27.4%) with hypertension history currently received anti-hypertensive therapy, and which was associated with lower systolic blood pressure (SBP) and diastolic blood pressure (DBP) on admission (SBP, *p* = 0.008; DBP, *p* = 0.017), less hematoma volume (9.8 vs. 11%, *p* = 0.006), and lower all-cause mortality at 3 months (15.3 vs. 19.8%, OR = 0.728, *p* = 0.016). In multivariable analysis, adjusting for age, gender, residence, ischemic stroke history, admission SBP and DBP, and current use of antihypertension were significantly associated with lower adjusted hazard ratios (HRs) for all-cause mortality at discharge (adjusted HR, 0.497, *p* = 0.012), 30 days (adjusted HR, 0.712, *p* = 0.015), and 90 days (adjusted HR, 0.766, *p* = 0.030). However, after adjusting the variable of hematoma volume, the mortality between the two groups was not significantly different.

**Conclusions:**

Preonset anti-hypertensive therapy was associated with lower mortality of ICH, which somewhat depended on hematoma volume.

## Introduction

Spontaneous intracerebral hemorrhage (ICH) is one of the most devastating diseases in the world with high morbidity and mortality, but without effective and specific treatment (1, 2). Recently, there are a series of studies about ICH therapies, including hematoma evacuation (3), hemostasis (4), and intensive BP reduction (5), but the outcomes are not satisfactory. Intensive BP reduction might prevent enlargement of hematoma and does not increase the ischemic area surrounding the hematoma (6). However, a series of randomized controlled trials failed to confirm that early intensive reduction of BP could reduce the mortality or major disability of patients with ICH at 90 days (7). Recently, Li et al. have reported that the BP reduction within 2 h attenuates hematoma growth and improves outcome in ICH, but the narrow therapeutic time window greatly limits the application (8). Thus, the preceding intervention of ICH may be a better strategy.

Hypertension is the most important independent risk factor for the development of ICH. Unfortunately, in China nearly half of adults aged 35–75 years have hypertension. However, only 30.1% of patients with hypertension are being treated and 7.2% are under control (9). Patients with untreated hypertension would be at a higher risk for the development of ICH, but whether preonset anti-hypertensive therapy could affect the morality and outcome of ICH remains unknown.

The objective of this study was to examine the hypothesis that preonset anti-hypertensive therapy could significantly affect the mortality and poor outcome of ICH.

## Materials and Methods

### Study Oversight

The CHEERY study was a large, multicenter cohort study in China and registered in the Chinese Clinical Trial Registry (No. ChiCTR 1900020872, http://www.chictr.org.cn). The CHEERY study aimed to establish a multicenter cohort of patients who suffered from ICH in China and to identify any major risk factors affecting the prognosis of ICH. During the study period (2018/12/1 to-2020/11/30), 31 recruitment sites jointly completed this study, and 3,460 consecutive patients were enrolled.

### Ethics Statement and Consent to Participates

The study protocol and data collection were conducted strictly following the Declaration of Helsinki and were approved by the Research Ethics Committee of Tongji Medical College, Huazhong University of Science and Technology, Wuhan, China (ethical approval number:2018-S485). Protocol training was conducted for all researchers prior to the implementation of the project. All participants signed a written informed consent prior to the enrollment.

### Study Sample or Population

Patients who were aged ≥18 years and had first-ever spontaneous ICH confirmed by computed tomography (CT) were recruited in the study. Eligible cases were selected if they presented within 7 days after symptom onset and had a previous diagnosis of hypertension *via* physicians, based on repeated office BP measurements or out-of-office BP measurement with daytime ambulatory BP monitoring (ABPM) and/or home BP monitoring (HBPM) according to hospital records and interviews from patients' relatives and family doctors. Moreover, antihypertensive drugs precede the onset of ICH were recorded as well. ICH cases due to secondary causes, which include trauma, primary subarachnoid hemorrhage, hemorrhagic conversion from ischemic stroke, and bleeding attributing to thrombolysis, were not eligible for the study. Clinical assessments and initial diagnoses were performed by clinical fellows and reviewed by a stroke neurologist.

### Measures

Demographics, medical histories, and hospitalization information were collected as follows: age, gender, residence, education, cigarette and alcohol consumptions, past histories, duration from onset to enrollment, ambulance use, SBP, and DBP. Medical history is based on all available information from patients, hospital records, and general practitioners. Hypertension is defined on the basis of the recommendation of the European Society of Cardiology (ESC) and the European Society of Hypertension (ESH) guidelines for the management of arterial hypertension (10). The most widely used diagnostic criteria were office SBP values ≥140 mmHg and/or DBP values ≥90 mmHg. Three BP measurements (1–2 min apart, in a resting state) were performed in both upper arms using a method of auscultatory or automatic sphygmomanometers in the doctor's office, and BP is recorded as the average of the last two BP readings. Other diagnostic criteria of hypertension include BP values ≥135/85 mmHg for 3–6 days average of HBPM, and ≥130/80 mmHg over 24 h, ≥135/85 mmHg for the daytime average, and ≥120/70 for the nighttime average of ABPM. The current antihypertension users were defined as patients who took monotherapy or drugs combination therapy with five major drug classes [angiotensin-converting enzyme (ACE) inhibitors, angiotensin receptor blockers (ARBs), beta-blockers, calcium channel blockers (CCBs), and diuretics] according to their last prescription 30 days before hospitalization for ICH. The baseline neurological deficits were assessed using the National Institutes of Health Stroke Scale (NIHSS) and the Glasgow Coma Scale (GCS). The features of intracerebral hematoma were analyzed on the initial CT scan. Hematoma localization was recorded, and the volume was calculated using the ABC/2 formula. Furthermore, intraventricular hemorrhage (IVH) was also recorded separately, for lack of hematoma volume.

The primary outcome was mortality at discharge and at 30 and 90 days. The secondary outcome was a poor functional outcome defined as the modified Rankin Scale (mRS) score of 4–6 at the same three-time points, representing severe functional dependence or death. We obtained discharge outcome data from the hospital registry system and followed patients with telephone or message interviews for information at 30 and 90 days after the onset of ICH.

### Statistical Analysis

In the statistical analyses, categorical variables were represented as number of cases with percentage, Pearson's chi-squared tests or Fisher's exact tests were used for comparison, as appropriate. Continuous variables were expressed as the mean ± SD (x ± SD) or median with interquartile range, and compared using the Mann–Whitney *U*-test. Multivariate Cox regression analyses in relation to mortality at discharge and at 30 and 90 days were carried out, and missing outcomes of 180 patients were processed as censored data. Age, gender, residence, history of ischemic stroke, admission SBP/DBP, hematoma location, presence of IVH, and hematoma volume were incorporated as required in three different hazard models. Hazard ratio (HR) and 95% CI were calculated after adjusting for covariates. All statistical tests were two-tailed, and *p* < 0.05 was considered to be statistically significant. All statistical analyses were performed using the IBM SPSS statistical software (version 26.0, IBM Corp., Armonk, NY, USA).

## Results

### Characteristics of Study Participants

**Figure 1:**
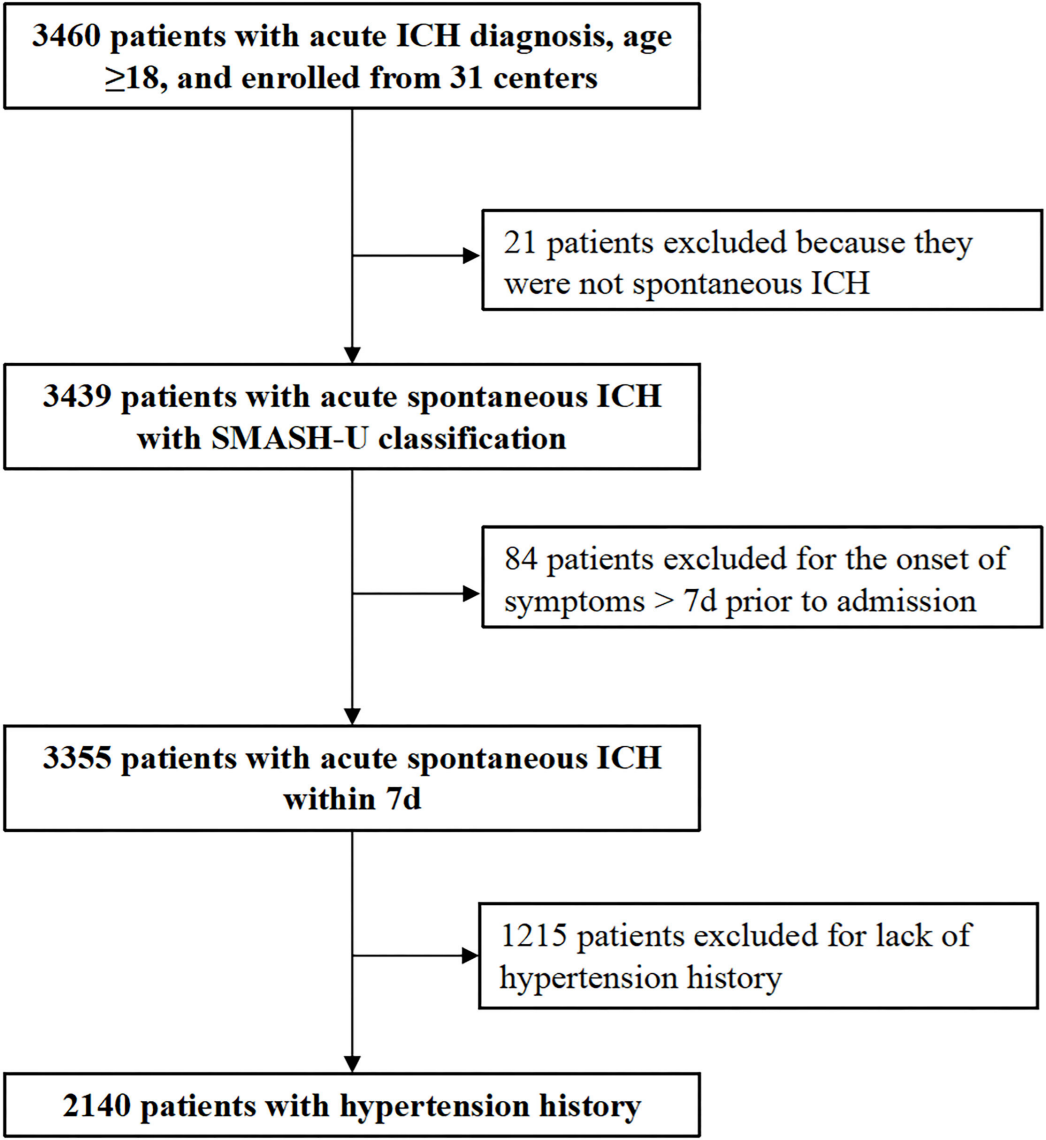
Flowchart of patient recruitment.

Between December 1, 2018 and November 30, 2020, a total of 3,460 consecutive patients with first-ever acute ICH were enrolled into the CHERRY study. After excluding 21patients who were not spontaneous ICH, 84 patients who presented >7 days after onset, 1,215 patients without hypertension history, and 2,140 patients (61.8%) with hypertension history were entered into the analysis. Among them, 587 patients (27.4%) were taking antihypertensive agents currently before the symptom onset (Figure 1 and Table 1 for detailed demographic information). The mean age was 62.4 (SD, 11.1) years in the current users, and 61.5 (SD, 11.9) years in the non-users (*p* = 0.152).

**Table 1:** Demographic and clinical characteristics of the participants.

**Characteristics**	**Total cohort**	**Current use**	**Non-use**	***p-*value**
**Total No**.	**2,140**	**587 (27.4)**	**1,553 (72.6)**	
Gender				
Male	1,407 (65.7)	368 (62.7)	1,039 (66.9)	0.067
Female	733 (34.3)	219 (37.3)	514 (33.1)	
Age, x ± SD	61.8 ± 11.7	62.4 ± 11.1	61.5 ± 11.9	0.152
Residence				
Urban	934 (43.6)	286 (48.7)	648 (41.7)	0.012^*^
Rural	1,183 (55.3)	294 (50.1)	889 (57.2)	
Unknown	23 (1.1)	7 (1.2)	16 (1.0)	
Smoking	636 (29.7)	167 (28.4)	469 (30.2)	0.4
Alcohol use	520 (24.3)	147 (25.0)	373 (24.0)	0.622
Pre-ICH Medical History				
Ischemic heart disease	96 (4.5)	32 (5.5)	64 (4.1)	0.185
Diabetes	241 (11.3)	73 (12.4)	168 (10.8)	0.291
Ischemic stroke	256 (12.0)	96 (16.4)	160 (10.3)	<0.001^*^
Hyperlipidemia	77 (3.6)	26 (4.4)	51 (3.3)	0.204
Hematologic disorders	7 (0.3)	3 (0.5)	4 (0.3)	0.401
Thyroid disease	17 (0.8)	5 (0.9)	12 (0.8)	0.791
Neoplastic disease	29 (1.4)	11 (1.9)	18 (1.2)	0.202
Duration from onset to enrollment				
≤ 6 h	1,253 (58.6)	353 (60.1)	900 (58.0)	0.499
7–24 h	546 (25.5)	144 (24.5)	402 (25.9)	
25–72 h	236 (11)	67 (11.4)	169 (10.9)	
4–7 days	105 (4.9)	23 (3.9)	82 (5.3)	
Admission SBP (mmHg)	168 (151–188)	167 (150–181)	170 (152–190)	0.008^*^
Admission DBP (mmHg)	97 (87–109)	96 (86–106)	98 (88–110)	0.017^*^
Admission NIHSS	9 (4–15)	9 (3–15)	9 (4–16)	0.551
Admission GCS	14 (11–15)	14 (12–15)	14 (10–15)	0.106
Hematoma volume^b^	10.1 (5–23.2)	9.8 (4–20)	11 (5–24.4)	0.006^*^
Location of hematoma				
Supratentorial	1,757 (82.1)	478 (81.4)	1,279 (82.4)	0.417
Infratentorial	274 (12.8)	81 (13.8)	193 (12.4)	
IVH	328 (15.3)	96 (16.4)	232 (14.9)	0.417

* *p < 0.05*.

a *Excluding patients with IVH*.

*DBP, diastolic blood pressure; GCS, Glasgow Coma Scale; ICH, intracerebral hemorrhage; IVH, intraventricular hemorrhage; NIHSS, National Institutes of Health Stroke Scale; NO., number; SBP, systolic blood pressure*.

Univariate testing showed patients with ICH who currently take antihypertensive drugs were more likely to live in an urban area (48.7 vs. 41.7%, *p* = 0.012) and have ischemic stroke history (16.4 vs. 10.3%, *p* < 0.001). Their SBP and DBP on admission were both lower (SBP, *p* = 0.008; DBP, *p* = 0.017) than those who did not currently take antihypertensive drugs. After excluding patients with IVH whose hematoma volume could not be well estimated, we found patients who currently take antihypertensive drugs had less hematoma volume than those non-users (9.8 vs. 11%, *p* = 0.006) (Table 1).

### Association of Currently Taking Antihypertensive Drugs With Outcomes of ICH

Clinical outcomes of patients with ICH were evaluated by mRS score at discharge and at 30 and 90 days after ICH onset. Univariate testing showed that the dichotomized change in mRS score of patients with ICH who currently take antihypertensive drugs was not different from those non-users after ICH onset. However, their all-cause death was lower than those non-users at discharge (2.7 vs. 5.2%, OR = 0.516, 95% CI, 0.299–0.890; *p* = 0.016), 30 days (11.6 vs. 16.2%, OR = 0.68, 95% CI, 0.510–0.908; *p* = 0.009), and 90 days (15.3 vs. 19.8%, OR = 0.728, 95% CI, 0.561–0.943; *p* = 0.016) after ICH onset (Table 2).

**Table 2:** Clinical outcomes of the participants.

**Outcome**	**Current use**	**Non-use**	***p-*value**	**OR**	**95% CI**
Discharge mRS					
0–3	297/582 (51.0)	823/1,530 (53.8)	0.256	1.117	0.923–1.352
4–6	285/582 (49.0)	707/1,530 (46.2)			
30-day mRS					
0–3	313/577 (54.2)	819/1,532 (53.5)	0.747	0.969	0.800–1.174
4–6	264/577 (45.8)	713/1,532 (46.5)			
90-day mRS					
0–3	373/577 (64.7)	963/1,528 (63.0)	0.491	0.932	0.763–1.138
4–6	204/577 (35.3)	565/1,528 (37.0)			
Discharge death	16/587 (2.7)	80/1,553 (5.2)	0.016^*^	0.516	0.299–0.890
30-day death	67/577 (11.6)	248/1,532 (16.2)	0.009^*^	0.68	0.510–0.908
90-day death	88/577 (15.3)	303/1,528 (19.8)	0.016^*^	0.728	0.561–0.943

* *p < 0.05*.

*mRS, modified Rankin Scale; OR, odds ratio*.

### Multivariable Analysis

**Figure 2:**
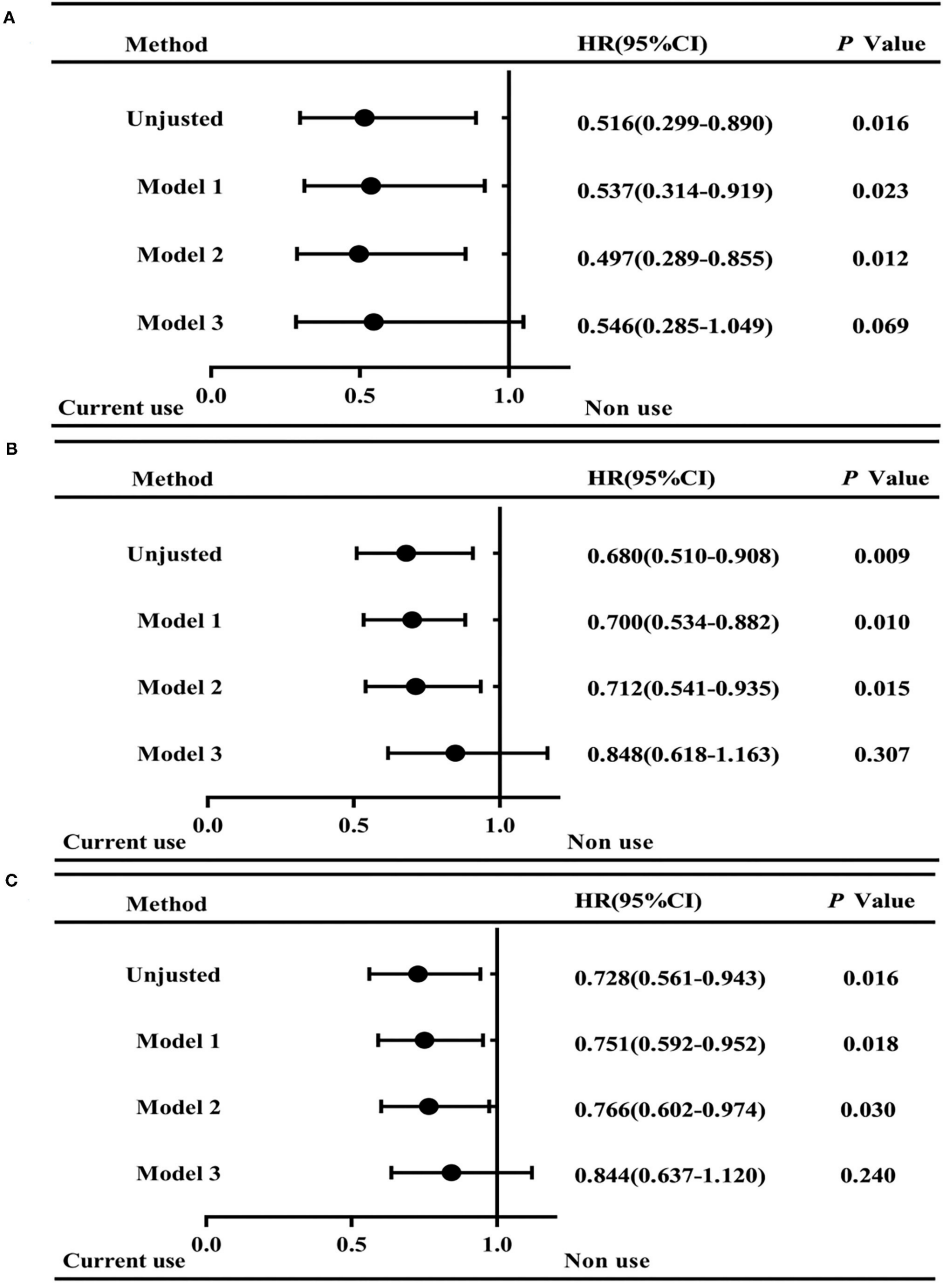
Hazard ratios (95%CI) for mortality in ICH patients of current antihypertension drug use and non-use. Graphs show forest plots for HR for all-cause mortality at discharge **(A)**, 30 days **(B)**, and 90 days **(C)** after ICH onset. Model 1: Adjusted for age and gender. Model 2: Adjusted for age, gender, residence, ischemic stroke history, admission SBP and DBP. Model 3: Adjusted for age, gender, residence, ischemic stroke history, admission SBP and DBP, and hematoma volume. HR, hazard ratio. Non-use group is as the Reference. *p < 0.05.

For all-cause death, the following variables were used in the adjusted models: age (as a continuous variable), gender, residence, ischemic stroke history, admission SBP and DBP, and hematoma volume. Results showed that currently taking antihypertensive drugs was associated with lower adjusted HR for discharge mortality than those non-users (adjusted HR, 0.497; 95% CI, 0.289–0.855; *p* = 0.012), apart from adjusting the variable of hematoma volume (Figure 2A). In addition, the mortality at 30 and 90 days after ICH onset had a similar trend (30 days: adjusted HR, 0.712, 95% CI, 0.541–0.935; *p* = 0.015; and 90 days: adjusted HR, 0.766, 95% CI, 0.602–0.974; *p* = 0.030) (Figures 2B,C). However, after adjusting the variable of hematoma volume, the mortality between the two groups was not significantly different (Figure 2).

## Discussion

In this large-scale, multicenter cohort of patients with ICH, we found that patients of acute spontaneous ICH with hypertension was as high as 61.8%, but only 27.4% among them currently received antihypertensive therapy. The current use of antihypertensive drugs prior to the ICH onset was associated with lower SBP and DBP on admission, less hematoma volume and lower all-cause mortality. In multivariable analysis, adjusting for age, gender, residence, ischemic stroke history, admission SBP and DBP, and current use of antihypertensive medication was significantly associated with lower adjusted HRs for all-cause mortality at discharge and at 30 and 90 days after ICH onset. However, after adjusting the variable of hematoma volume, the mortality between the two groups was not significantly different.

The ICH accounts for 10 to 15% of all strokes and 50% of stroke-related mortality. Acute ICH is a life-threatening illness of global importance and causes more than 2 million deaths worldwide each year (11, 12). The 2017 data showed that the incidence and prevalence of ICH were 23.8 and 15.8%, respectively, in China. The burden of ICH was significantly higher than that of other developing countries (9). Hypertension is the most important but a treatable risk factor for the prevention of spontaneous ICH. At present, the prevalence of hypertension in China is 23.8%, about 200 million patients. However, only 30.1% of patients with hypertension are being treated, and 7.2% of them are under control (9). In this ICH cohort, 27.4% of patients received antihypertensive treatment prior to the ICH onset, and the SBP and DBP levels were lower in patients who currently take antihypertensive drugs than in those non-users.

It is noted that even in the current use group, the BP on admission was higher than the normal level. We speculated that this is on the one hand because of the lack of the hypertension control, on the other hand, because of the acute hypertensive response (AHR), which is a common systemic response to the occurrence of ICH. AHR may occur frequently and of a greater magnitude in those with chronic hypertension, and is associated with hematoma expansion and increased mortality (13).

Studies examining the association between BP reduction and prognosis of patients with an acute ICH have mostly targeted at the acute intervention or treatment after ICH. The Intensive Blood Pressure Reduction in Acute Cerebral Hemorrhage Trial (INTERACT2) conducted intensive SBP reduction (target <140 mmHg within 1 h, cessation of treatment at <130 mmHg) within 6 h of onset of ICH (14), and the second Antihypertensive Treatment of Acute Cerebral Hemorrhage Trial (ATACH-II) with 1,000 patients conducted a more intensive strategy (target 110–139 mmHg within 2 h) within 4.5 h of symptom onset (15). These two large trials have shown inconsistent results: INTERACT 2 found that intensive SBP reduction did not improve death or major disability at 90 days, but improved functional recovery on a number of secondary outcomes. ATACH-II reported no benefit but an excess of adverse renal events. Subgroup analysis in ATACH-II showed that intensive BP reduction within ≤ 2 h after onset of symptoms by giving intravenous nicardipine, reduced hematoma growth, and improved functional outcome (8). In this prior study, we have found that the current use of antihypertensive medication prior to the onset of ICH had lower hematoma volume than those non-users, which may be associated with the lower BP on admission. This needs further research.

For the clinical outcomes, univariate analysis showed a significant difference between antihypertensive medication users and non-users, and when adjusting for age, gender, residence, ischemic stroke history, and admission SBP and DBP, a significant trend of lower mortality was still observed in the current use group. In our study, both short-term mortality (at discharge) and long-term (at 90 days) mortality were lower in the patients who received antihypertensive treatment prior to the ICH onset. However, after adjusting the variable of hematoma volume, the mortality between the two groups was not significantly different. It indicated that hematoma volume may play a role in the association between BP control preceding the onset of ICH and the mortality of ICH. Specifically, preonset antihypertensive treatment may reduce the mortality after ICH by mediating hematoma volumes, which was similar to previous findings reporting that hematoma volume is a powerful neuroimaging predictor of 30-day mortality in ICH (16) and high burden of BP-related alleles are associated with larger baseline hematoma volume and poor clinical outcome after ICH (17). In this study, BP control preceding the onset of ICH was associated with lower BP, smaller hematoma volume, and lower mortality, indicating that strategies to control BP preceding ICH onset, even for a short-term, such as 1 month as in this study, could be beneficial to ICH.

In this study, the mortality was lower than that in previous studies, which may attribute to the following reasons. On one hand, this study only included hospitalized patients, and those who were treated in the outpatient setting and died before reaching the hospital were not included; on the other hand, this cohort study spanned the period of the COVID-19 epidemic in time. During this period, many patients with ICH combined with COVID-19 infection were isolated (due to policy reasons), and these patients were not included in this study.

The strengths of our study included large sample size and multicenter design. Furthermore, we focused on the use of antihypertensive medication prior to the onset of ICH. Our study also has several limitations and should be interpreted with caution. First, this study was a retrospective cohort study, and the selection bias may be present. Only hospitalized patients were included in this study, and those patients who could not afford to go to hospital or died before reaching the hospital were not included. Second, for this is a study about Chinese patients, who have different genetic background and dietary habits compared with those from Western countries, caution should be taken in generalizing the findings. Third, in this study, we used the old definition for hypertension, which was different from the newest one. However, considering the medical background of the patients at the beginning of the study, we did not analyze the data according to the newest definition. Lastly, whether the BP was controlled in the current users and reasons for non-current use of antihypertension in patients with hypertension were not analyzed in this study.

In a word, we propose that preonset anti-hypertensive therapy was associated with lower mortality of ICH, which somewhat dependent on hematoma volume.

## Data Availability Statement

The raw data supporting the conclusions of this article will be made available by the authors, without undue reservation.

## Ethics Statement

The studies involving human participants were reviewed and approved by the Research Ethics Committee of Tongji Medical College, Huazhong University of Science and Technology, Wuhan, China (ethical approval number:2018-S485). The patients/participants provided their written informed consent to participate in this study. Written informed consent was obtained from the individual(s) for the publication of any potentially identifiable images or data included in this article.

## Author Contributions

BH was responsible for the concept and design of the study. YW and QH did the literature search and wrote the manuscript. SC, ML, YX, LZ, ZS, and XC acquired and interpreted data. HG and JS analyzed the data. DW revised the manuscript. JC did the administrative, technical, or statistical support. All authors contributed to the article and approved the submitted version.

## Funding

This work was supported by the National Key Research and Development Program of China (No. 2018YFC1312200 to BH) and the National Natural Science Foundation of China (Nos. 81820108010 to BH, 81901214 to YW, and 82071335 to QH). All sources of funding received for the research have been submitted.

## Conflict of Interest

The authors declare that the research was conducted in the absence of any commercial or financial relationships that could be construed as a potential conflict of interest.

## Publisher's Note

All claims expressed in this article are solely those of the authors and do not necessarily represent those of their affiliated organizations, or those of the publisher, the editors and the reviewers. Any product that may be evaluated in this article, or claim that may be made by its manufacturer, is not guaranteed or endorsed by the publisher.

## Abbreviations

AHR: acute hypertensive response
ATACH-II: The Second Antihypertensive Treatment of Acute Cerebral Hemorrhage Trial
CHEERY: Chinese cerebral hemorrhage: mechanism and intervention study
DBP: diastolic blood pressure
GCS: the Glasgow Coma Scale
HR: hazard ratio
IVH: intraventricular hemorrhage
INTERACT2: The Intensive Blood Pressure Reduction in Acute Cerebral Hemorrhage Trial
mRS: modified Rankin Scale
NIHSS: National Institutes of Health Stroke Scale
OR: odds ratio
SBP: systolic blood pressure.
